# Food consumption of adults in Germany: results of the German National Nutrition Survey II based on diet history interviews

**DOI:** 10.1017/S0007114515000744

**Published:** 2015-04-13

**Authors:** Thorsten Heuer, Carolin Krems, Kilson Moon, Christine Brombach, Ingrid Hoffmann

**Affiliations:** 1 Department of Nutritional Behaviour, Max Rubner-Institut, Federal Research Institute of Nutrition and Food, Haid-und-Neu-Straße 9, 76131Karlsruhe, Germany; 2 Institute for Food and Beverage Innovation, Centre for Nutrition, Zurich University of Applied Sciences, Einsiedlerstrasse 34, 8820Wädenswil, Switzerland

**Keywords:** Food consumption, Adults, Socio-economic status, Diet history interviews, German National Nutrition Survey II

## Abstract

The second German National Nutrition Survey (NVS II) aimed to evaluate food consumption and other aspects of nutritional behaviour of a representative sample of the German population, using a modular design with three different dietary assessment methods. To assess usual food consumption, 15 371 German speaking subjects 14–80 years of age completed a diet history interview between November 2005 and November 2006. With reference to the guidelines of the German Nutrition Society (DGE), NVS II observed that the German population did not eat enough foods of plant origin, especially vegetables and consumed too much of meat and meat products. While generally similar food consumption is observed in other European countries, consumption of bread, fruit juices/nectars and beer is higher in Germany. On average, men consumed two times more meat and soft drinks as well as six times more beer than women did, whereas the consumption of vegetables, fruit as well as herbal/fruit tea was higher in women. Older participants showed a lower consumption of meat, fruit juice/nectars, soft drinks and spirits as well as a higher consumption of fish, vegetables, fruit, and herbal/fruit tea than adolescents and younger adults did. There are also differences in food consumption with regard to socio-economic status (SES). Persons with higher SES consumed more vegetables, fruit, fish, water, coffee/tea and wine, while persons with lower SES consumed more meat and meat products, soft drinks and beer. In general, the food consumption of women, the elderly and the higher SES group tends to be closer to the official dietary guidelines in Germany.

National nutrition surveys provide information on food consumption and nutritional behaviour of the general population and specific population groups, such as age groups and socio-economic status (SES) groups. These data serve amongst others as a basis for national and international dietary guidelines, for scientific issues as well as for decision- and policy-making.

Since the first German National Nutrition Survey, carried out from 1985 till 1988^(^ ^1^ ^)^, living conditions and life style of many people in Germany have changed as the political situation (reunion) and food supply. Against this background, the Federal Ministry of Food, Agriculture and Consumer Protection commissioned the Max Rubner-Institut to conduct the German National Nutrition Survey II (NVS II) to provide current, reliable and representative data on food consumption and further aspects of nutritional behaviour of the German population. The NVS II shows a modular design by applying three dietary assessment methods to meet different demands of a dietary survey, e.g. nutritional behaviour research or risk assessment. The present article provides results on food consumption of adults and adolescents living in Germany and of specific subgroups (sex, age and SES) of the NVS II based on diet history interviews. For a comprehensive overview of food consumption behaviour of the German population the most important sociodemographic parameters were considered. By using the diet history approach, usual food consumption patterns are described and compared with the dietary guidelines of the German Nutrition Society (DGE)^(^ ^2^ ^)^. Furthermore, the results are compared with those of food consumption surveys in other European countries.

## Methods

### Study design

The NVS II is a nationwide representative study conducted in Germany between November 2005 and January 2007. The participants were 14–80 years of age, German speaking and living in private households.

In the NVS II a two-stage sampling procedure was used. In the first stage all municipalities in Germany were stratified by administrative district and type (e.g. rural and urban). With reference to this stratification matrix, 500 nationwide sample points were randomly identified from the total number of municipalities in Germany, considering the proportion of the population living in each federal state and administrative district. At each of the selected sample points, the required number of addresses was randomly drawn from the respective local population registries, stratified by sex and age. The complete pool of addresses was drawn at the same time before the beginning of the interviews. Subjects who refused to participate in the study were not replaced. The pool of drawn addresses was found adequate to achieve the target number of 20 000 participants. The NVS II was approved by the German Federal Data Protection Office. Respondents were informed in detail about the study objectives, interview and examination procedures as well as the handling of data records and analyses under pseudonymous conditions. It was made clear that participation was on a voluntary basis and could be terminated at any time. In total, 46 587 individuals were contacted by an invitation letter and invited to a study centre. Across Germany 19 329 subjects agreed to participate. The response rate was 42 %.

**Fig. 1:**
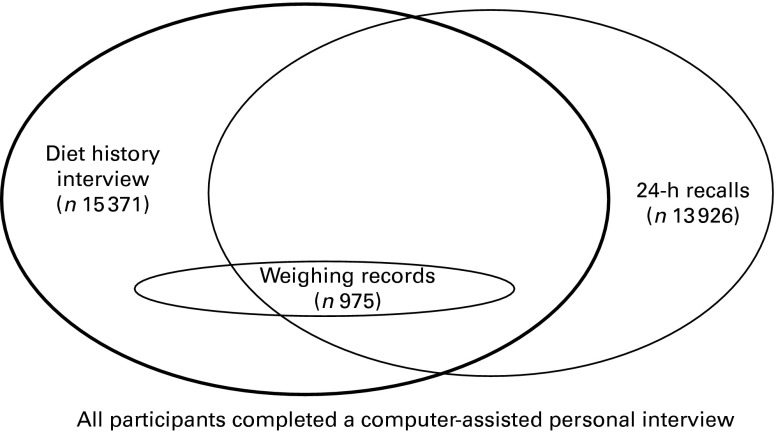
Dietary assessment methods applied in the second German National Nutrition Survey.

Figure 1 provides an overview of the dietary assessment methods applied in the NVS II and of the corresponding number of participants. A computer-assisted personal interview (*n* 19 329) and a complementary self-administered questionnaire (*n* 14 288) were the instruments used to obtain information on sociodemographics, such as education, occupation, household structure and income as well as nutritional behaviour, health aspects (e.g. smoking), use of dietary supplements, food purchase, leisure time activities and sleeping behaviour. Anthropometric measurements (body height and body weight, *n* 14 331) were determined according to the technique described by Lohman and colleagues^(^ ^3^ ^)^. Height was measured with portable Harpenden Stadiometers (Holtain Limited) and body weight with the calibrated scale seca 862 (seca Vogel & Halke) after shoes, coats and sweaters had been taken off. BMI was calculated using the following formula:
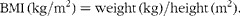

The usual food consumption of 15 371 participants of the study was assessed from November 2005 to November 2006 through a personal diet history interview using the programme DISHES (Diet Interview Software for Health Examination Studies). DISHES 98 was developed and validated by the Robert Koch-Institute in Germany and applied in the Nutrition Module of the German National Health Interview and Examination Survey of 1998^(^ ^4^ ^,^ ^5^ ^)^. DISHES 2005 was used for NVS II after it was adapted to the requirements of the present study^(^ ^6^ ^)^.

During the DISHES interview at the study centres participants were asked to give details of the foods and beverages they consumed during the preceding 4 weeks. First, information on usual meal patterns was obtained. Using this information, frequency and consumed amounts of individual food items or beverages of each meal occasion were assessed next in a standardised manner. For quantification of consumed amounts, tableware models (cups, glasses, spoons, plates and bowls) and an excerpt of the EPIC-SOFT picture book with different portion sizes of food items were used^(^ ^7^ ^,^ ^8^ ^)^. Food items in the DISHES programme are directly linked with the German nutrient database (BLS) which allows calculation of energy and nutrient intake and splitting up of recipes into ingredients^(^ ^9^ ^)^. In order to enhance data quality, high attention was paid to quality assurance procedures. Therefore, data controls for plausibility were performed to identify and correct data input errors (e.g. for quantities, extremes in energy and nutrient intake) and about 3400 interviewer's comments were checked and integrated into the interview data.

To quantify ingredients of composite dishes (e.g. meat in a lasagne), recipes which were named by the participants in the diet history interviews were disaggregated into their ingredients except for bread, pastries, soups, sauces and confectionery. The disaggregation is based on the recipes of the German Nutrient Database (BLS version 3.01)^(^ ^9^ ^)^. Thereafter all foods were categorised into food groups (Table 1).

**Table 1:** Description of the food groups

Food group	Foods included
Bread	Including rolls, baguette, toast, rusk
Cereals and cereal products	Cereals, flours, rice and processed products like breakfast cereals, pasta, popcorn
Potatoes and potato products	Fresh potatoes, heated and processed products like chips, potato pancakes, potato crisps
Pastries	Cake, pies, piquant pastries like filled puff paste, cheese straw, snacks, peanut flips, cracker, tortilla chips
Vegetables, vegetable products, mushrooms and pulses	
Raw	Including frozen vegetables
Heated	Including vegetable products
Fruit and fruit products (without juice)	
Fruit	Including unsweetened frozen fruit
Fruit products	Including fruit sauces and processed products like sweetened or heated fruit, canned fruit, dried fruit
Nuts and seeds	Hazelnuts, peanuts, almonds etc. or processed products like peanut butter, salted or roasted nuts
Milk, dairy products and cheese	
Milk and mixed milk drinks	Including cacao drinks, milkshakes
Dairy products	Yoghurt, (sour) cream, buttermilk, kefir, whey
Cheese and curd cheese	Including soft cheese, cream cheese, acid curd cheese
Meat, meat products and sausages	Meat products: including meat sauces and processed meat products like cured pork, meatballs
Fish, fish products and seafood	Salt- and freshwater fish, shrimps, mussels, snails, processed products like caviar, tinned fish
Eggs	
Fats and oils	
Vegetable fats and oils	Including margarine and other spreadable fats
Animal fats	Including butter
Soups	
Sauces and spicy ingredients	Warm and cold sauces (e.g. ketchup, fruit sauces), mustard, vinegar etc.
Confectionery, total	
Sweets, ice cream and desserts	Sweets, ice cream, cream, desserts, powdered drinks, - granulate
Sweet spreads	Jam, jellies, honey and chocolate spreads
Sweetener	Sweeteners, sugar substitutes
Non-alcoholic beverages	Water, coffee and tea (black/green), herbal/fruit tea, fruit juices/nectars, soft drinks, other non-alcoholic beverages: malt coffee, malt beer, non-alcoholic beer/sparkling wine
Alcoholic beverages	Beer (including mixed beer drinks), wine and sparkling wine, spirits and other alcoholic beverages: schnapps, liqueurs, cocktails, alcopops

### Statistical analysis

Regarding demographical characteristics, the sample of the NVS II participants who completed the diet history interviews differs only slightly from the total population of Germany assessed by the ‘Microcensus 2006’ which provides official representative statistics of the population in Germany^(^ ^10^ ^,^ ^11^ ^)^. To ensure representativeness of the German population, existing differences were compensated by weighting the data according to ‘Microcensus 2006’ for gender, age, federal state, administrative district, school education, employment and household size. Furthermore, data were weighted on an equal distribution of the interview month over the study period.

To describe the social status of participants, a social class-index was created based on net monthly income of the household, school education level of the participant and employment status of the principal earner of the household. Based on this index participants were assigned to five social classes: upper, higher middle, middle, lower middle and lower class^(^ ^12^ ^)^. For reasons of clarity and readability, the social classes were aggregated to three classes: upper ( = upper and higher middle), middle as well as lower ( = lower middle and lower) class.

Underreporting of participants was calculated via ratio of energy intake and resting energy expenditure. Energy intake was calculated using the BLS version 3.01^(^ ^9^ ^)^. Resting energy expenditure was calculated by the use of the formula of Müller *et al.* ^(^ ^13^ ^)^, considering body height and body weight. Underreporting was defined as ratio of energy intake and resting energy expenditure < 1·09 which was calculated using equations derived by Goldberg *et al.* ^(^ ^14^ ^)^, and adopted by Black^(^ ^15^ ^)^.

Food consumption is shown as arithmetic mean and 95 % CI. Comparisons are made with the CI of the mean. Differences between groups are considered to be significant if CIs do not overlap. Arithmetic mean for food consumption was chosen because foods eaten rarely (e.g. nuts and seeds) were consumed by less than 50 % of the participants, leading to medians with the value ‘0’. This is of restricted use when comparing food consumption. All statistical analyses were performed using the statistical software package SAS version 9.2 (SAS Institute, Inc.).

## Results

Of the 15 371 participants of the NVS II who completed the diet history interview, 7093 were men (46·1 %) and 8278 women (53·9 %). Mean age was 46·3 years for men and 46·1 years for women with an age range from 14 to 80 years. The study population of adult men and women was on average overweight (BMI>25 kg/m^2^) and nearly 30 % of the participants were smokers (Table 2). Approximately a quarter of the participants had completed higher education (12 or 13 years of school education), and more than half were employed.

**Table 2:** Description of the NVS II (German National Nutrition Survey II) participants who completed the diet history interview (*n* 15 371)* (Mean values with their standard errors; percentages)

	Men (*n* 7093)	Women (*n* 8278)
Age group (%)		
14–18 years	7·2	6·6
19–24 years	8·8	7·9
25–34 years	14·5	14·2
35–50 years	31·6	30·1
51–64 years	20·6	20·6
65–80 years	17·4	20·6
BMI† (kg/m^2^)		
14–18 years		
Mean	22·5	22·1
se	0·2	0·2
19-64 years		
Mean	26·7	25·6
se	0·1	0·1
65–80 years		
Mean	28·4	28·3
se	0·1	0·1
Smoker (%)	33·8	24·4
School education (%)		
Pupils	5·8	5·9
9 years	41·0	39·1
10 years	25·5	29·8
12 or 13 years	24·9	22·2
Other	2·9	3·0
Employees (%)	60·0	48·6
SES (%)		
Low	23·8	28·2
Medium	33·2	33·5
High	43·0	38·3

SES, socio-economic status.

* Data are weighted.

† 6 % of the participants (376 men and 566 women) did not take part in the anthropometric measurements.

### High-carbohydrate foods

Concerning the mean daily consumption of high-carbohydrate foods by age and sex, men of all age groups generally consumed more bread, cereals and cereal products, potatoes and potato products and pastries than women (Table 3). While the consumption of bread was similar between young and older subjects, the younger subjects (14–50 years) ate more cereals and cereal products and pastries than the older ones. Older subjects (65–80 years) consumed more potatoes and potato products than younger subjects. The DGE recommends overall 400–550 g of bread, cereals and cereal products and potatoes and potato products per d. Men's consumption was slightly below these dietary guideline values. Women, however, recorded a little less than three-fourth of the recommended amount.

**Table 3:** Mean daily consumption of high carbohydrate foods (g) by sex and age groups (Mean values and 95 % confidence intervals)

Food group	Sex	14–18 years	19–24 years	25–34 years	35–50 years	51–64 years	65–80 years	Average
Mean	95 % CI	Mean	95 % CI	Mean	95 % CI	Mean	95 % CI	Mean	95 % CI	Mean	95 % CI	Mean	95 % CI
Bread	Males	197	189, 205	183	172, 194	186	178, 195	190	185, 195	182	178, 187	171	167, 175	184	182, 187
	Females	148	142, 154	125	118, 131	133	128, 138	136	133, 139	136	133, 140	136	133, 139	136	134, 137
Cereals and cereal products	Males	101	95, 106	103	95, 110	100	95, 106	85	82, 88	62	60, 65	53	50, 55	80	78, 81
	Females	84	80, 88	89	83, 94	88	84, 92	76	74, 78	55	53, 57	46	44, 48	69	68, 70
Potatoes and potato products	Males	93	89, 97	101	95, 108	86	81, 91	90	87, 93	89	87, 92	106	103, 109	93	92, 95
	Females	68	65, 72	65	61, 69	70	66, 73	69	67, 70	72	70, 74	85	83, 87	73	72, 74
Pastries	Males	54	50, 58	50	46, 55	49	45, 52	45	43, 47	38	36, 40	35	33, 37	43	42, 44
	Females	36	33, 39	34	32, 37	36	34, 38	34	32, 35	30	29, 32	28	26, 29	32	32, 33

### Vegetables, fruit, nuts and seeds

Consumption of vegetables, vegetable products, mushrooms and pulses was on average slightly higher for women than for men (Table 4). Women consumed more raw vegetables, whereas men ate more heated products. Across the age groups an increasing consumption of total vegetables was observed for the age group of 51–64 years of both sexes. Of the different age groups, only the middle-aged women (35–64 years) showed a higher consumption of total vegetables compared to men of the same age group. Compared to the dietary guidelines of the DGE (400 g/d vegetables), men and women consumed about half of the recommended amount of vegetables.

**Table 4:** Mean daily consumption of vegetables, fruit, nuts and seeds (g) by sex and age groups (Mean values and 95 % confidence intervals)

Food group	Sex	14–18 years	19–24 years	25–34 years	35–50 years	51–64 years	65–80 years	Average
Mean	95 % CI	Mean	95 % CI	Mean	95 % CI	Mean	95 % CI	Mean	95 % CI	Mean	95 % CI	Mean	95 % CI
Vegetables, vegetable products, mushrooms and pulses	Males	187	177, 196	212	198, 225	222	211, 232	237	231, 244	245	238, 252	237	231, 244	231	227, 234
Females	196	187, 206	206	195, 218	232	223, 241	257	252, 263	260	253, 268	231	225, 238	241	238, 244
Raw	Males	97	90, 105	111	100, 122	113	106, 121	126	122, 131	135	129, 141	116	110, 121	121	118, 124
	Females	122	115, 129	113	104, 121	132	125, 139	151	146, 156	149	143, 155	120	115, 125	136	134, 139
Heated	Males	89	84, 94	101	94, 108	108	102, 114	111	108, 114	110	107, 113	122	118, 125	110	108, 112
	Females	74	70, 78	94	87, 100	100	95, 104	107	104, 109	112	108, 115	112	108, 115	105	103, 106
Fruit and fruit products (without juice)	Males	175	161, 190	161	146, 176	181	168, 193	218	209, 227	275	264, 287	299	287, 310	230	225, 235
Females	226	209, 242	212	196, 227	250	234, 265	260	252, 268	331	319, 343	318	307, 328	279	274, 284
Fruit	Males	172	157, 186	157	142, 172	176	164, 189	212	203, 221	267	256, 278	279	268, 291	222	217, 227
	Females	219	203, 235	209	193, 224	245	229, 260	253	246, 261	322	310, 334	299	289, 309	270	265, 275
Fruit products	Males	4	3, 5	4	3, 5	5	3, 6	6	5, 7	9	8, 10	19	17, 22	8	8, 9
	Females	7	5, 9	3	2, 4	5	4, 6	7	6, 8	9	8, 10	19	17, 20	9	9, 10
Nuts and seeds	Males	3	2, 4	3	3, 4	4	3, 5	6	5, 7	5	5, 6	3	3, 4	5	4, 5
	Females	2	1, 3	3	2, 3	3	2, 4	4	3, 4	5	5, 6	3	2, 3	4	3, 4

Women in general consumed more fruit and fruit products than men. The consumption was higher in older age groups (51–80 years) for both sexes. Women aged 25–80 years and men aged 51–80 years met the dietary guidelines of the DGE (at least 250 g/d fruits). Among all age groups, a small number of men and women consumed nuts and seeds resulting in small average amounts of consumption.

### Milk, dairy products and cheese

The consumption of milk, dairy products and cheese in general was higher in men than in women in the age groups 14–50 years (Table 5). However, there is no difference between both sexes in older age groups (51–80 years). Men consumed more milk and mixed milk drinks than women, except for the age group of 65 and older. Women aged 35–80 years ate more dairy products than men of the same age, but the consumption of boys aged 14–18 years was higher than that of girls. In general, both men and women consumed similar amounts of cheese and curd cheese; however, in the age group 19–34 years, the consumption was higher in men than in women. The mean overall consumption of milk, dairy products and cheese of men, except for the age groups 51–80 years, conformed to the dietary guidelines of the DGE (250–310 g/d milk and dairy products); the consumption of women in general was found to be slightly below these guideline values.

**Table 5:** Mean daily consumption of milk, dairy products and cheese (g) by sex and age groups (Mean values and 95 % confidence intervals)

Food group	Sex	14–18 years	19–24 years	25–34 years	35–50 years	51–64 years	65–80 years	Average
Mean	95 % CI	Mean	95 % CI	Mean	95 % CI	Mean	95 % CI	Mean	95 % CI	Mean	95 % CI	Mean	95 % CI
Milk, dairy products and cheese	Males	345	325, 365	305	275, 335	291	266, 316	256	244, 267	227	216, 239	219	207, 230	259	253, 266
	Females	250	234, 267	251	227, 275	243	231, 255	231	223, 239	237	227, 246	231	221, 240	237	232, 241
Milk and mixed milk drinks	Males	233	214, 252	191	163, 219	165	142, 188	128	118, 139	96	87, 106	87	78, 96	133	127, 139
	Females	156	140, 171	136	114, 159	119	110, 128	95	89, 101	79	72, 86	86	78, 94	100	97, 104
Dairy products	Males	75	67, 82	69	61, 78	80	72, 87	82	78, 87	81	75, 87	81	76, 87	80	77, 82
	Females	61	56, 66	80	72, 87	86	80, 92	92	88, 96	108	103, 113	96	90, 101	92	90, 95
Cheese and curd cheese	Males	37	35, 40	45	41, 50	46	42, 50	45	43, 47	50	47, 53	50	48, 53	46	45, 48
	Females	34	32, 37	34	32, 37	38	36, 40	44	43, 46	49	47, 51	49	47, 52	44	43, 45

### Meat, fish and eggs

Men ate almost twice as much meat, meat products and sausages than women (Table 6). Male and female seniors (65–80 years) consumed less meat, meat products and sausages than the other age groups. About 2·5 % of all participants did not eat meat, meat products and sausages during the preceding 4 weeks. Men in all age groups exceeded the dietary guidelines of the DGE (300–600 g/week = 43–86 g/d meat, meat products and sausages), whereas women were in the upper range of the guidelines.

**Table 6:** Mean daily consumption of meat, fish and eggs (g) by sex and age groups (Mean values and 95 % confidence intervals)

Food group	Sex	14–18 years	19–24 years	25–34 years	35–50 years	51–64 years	65–80 years	Average
Mean	95 % CI	Mean	95 % CI	Mean	95 % CI	Mean	95 % CI	Mean	95 % CI	Mean	95 % CI	Mean	95 % CI
Meat, meat products and sausages	Males	152	146, 159	177	167, 187	161	154, 168	150	146, 154	128	125, 132	105	102, 108	142	140, 144
	Females	84	80, 88	76	72, 81	84	81, 88	81	79, 83	74	72, 76	64	62, 66	76	75, 77
Meat and meat products	Males	89	84, 94	110	103, 117	101	97, 106	91	89, 94	78	76, 81	65	63, 67	87	86, 88
	Females	50	47, 53	50	47, 54	55	53, 58	54	53, 55	50	49, 52	42	40, 43	50	50, 51
Sausages	Males	63	59, 67	67	61, 72	60	55, 64	59	56, 61	50	48, 52	40	39, 42	55	54, 56
	Females	34	31, 36	26	24, 28	29	27, 31	27	36, 28	24	23, 25	22	21, 23	26	25, 27
Fish, fish products and seafood	Males	15	13, 16	22	19, 24	24	22, 26	28	27, 29	31	30, 33	33	32, 35	28	27, 28
	Females	11	10, 12	15	13, 17	19	17, 20	21	20, 22	27	26, 28	26	25, 27	22	21, 22
Eggs*	Males	16	15, 17	22	19, 24	20	18, 21	17	16, 18	16	15, 17	14	14, 15	17	17, 18
	Females	12	11, 13	11	10, 12	13	12, 14	13	12, 13	14	13, 14	11	11, 12	12	12, 13

* Without eggs in pastries, soups and sauces.

The consumption of fish was higher in men than in women. Older people (51–80 years) ate more fish than the younger ones. About 16 % of the participants declared that they did not eat fish, fish products and seafood in the preceding 4 weeks. The DGE recommends 150–220 g fish/week ( = 21–31 g/d). Men aged 14–18 years and women aged 14–34 years recorded values below the guidelines, while the other age groups met the guidelines.

With regard to the consumption of eggs men on average consumed more than women. The DGE recommends less than three eggs a week (including processed eggs). Assuming that an egg is about 60 g, the consumption of 180 g/week (26 g/d) should not be exceeded. The consumption of eggs among all age and sex groups was found short of these dietary guidelines, when eggs in pastries, soups and sauces are not included.

### Fats and oils

The daily consumption of fats and oils in total as well as of those of vegetable and animal origin viewed separately was generally higher in men than in women (Table 7). Men and women of all age groups showed no or only minor differences regarding the consumption of vegetable fats and oils *v.* animal fats. The consumption of both sexes is within the dietary guidelines of the DGE (25–45 g/d fats and oils).

**Table 7:** Mean daily consumption of fats and oils (g) by sex and age groups (Mean values and 95 % confidence intervals)

Food group	Sex	14–18 years	19–24 years	25–34 years	35–50 years	51–64 years	65–80 years	Average
Mean	95 % CI	Mean	95 % CI	Mean	95 % CI	Mean	95 % CI	Mean	95 % CI	Mean	95 % CI	Mean	95 % CI
Fats and oils*	Males	34	32, 36	34	31, 36	38	36, 41	39	38, 41	40	39, 41	39	37, 40	38	38, 39
	Females	23	22, 24	23	21, 25	25	24, 26	28	27, 28	28	27, 29	30	29, 31	27	27, 28
Vegetable fats and oils	Males	16	15, 17	17	16, 19	21	19, 22	20	19, 21	21	20, 22	19	18, 20	20	19, 20
	Females	12	11, 13	13	11, 14	14	13, 15	14	14, 15	15	14, 15	14	14, 15	14	14, 14
Animal fats	Males	18	16, 20	16	14, 18	17	16, 19	19	18, 21	19	18, 20	20	19, 21	19	18, 19
	Females	11	10, 12	10	9, 12	11	10, 12	14	13, 14	14	13, 14	16	15, 16	13	13, 14

* Without fats and oils in pastries, soups and sauces.

### Soups, sauces and confectionery

Of the remaining food groups, men on average consumed more soups than women, and seniors showed the highest consumption of soups (Table 8). In total and in all age groups, men consumed more sauces and spicy ingredients than women. The consumption of sweets, ice cream and desserts was slightly higher in men than in women and was higher in young and middle-aged participants (14–50 years) than in older ones. In general, the consumption of sweet spreads was slightly higher in men with the highest consumption in the older participants (65–80 years) of both sexes. The consumption of sweeteners was higher in men than in women.

**Table 8:** Mean daily consumption of soups, sauces and confectionery (g) by sex and age groups (Mean values and 95 % confidence intervals)

Food group	Sex	14–18 years	19–24 years	25–34 years	35–50 years	51–64 years	65–80 years	Average
Mean	95 % CI	Mean	95 % CI	Mean	95 % CI	Mean	95 % CI	Mean	95 % CI	Mean	95 % CI	Mean	95 % CI
Soups*	Males	50	44, 55	46	40, 53	55	49, 60	52	48, 55	54	50, 58	63	58, 68	54	52, 56
	Females	38	34, 42	48	41, 55	49	45, 54	45	42, 47	46	43, 49	55	51, 59	47	46, 49
Sauces and spicy ingredients	Males	45	42, 49	51	48, 55	47	44, 51	47	45, 48	43	42, 45	40	39, 42	45	44, 46
	Females	36	34, 39	36	34, 39	36	34, 38	35	34, 36	32	31, 33	31	30, 32	34	33, 35
Sweets, ice cream and desserts	Males	68	63, 73	54	49, 59	56	51, 61	49	47, 51	38	35, 40	32	30, 34	46	45, 48
	Females	61	57, 66	63	54, 72	55	51, 58	46	43, 48	32	30, 34	30	28, 32	43	42, 45
Sweet spreads	Males	20	18, 22	12	10, 14	16	14, 19	17	16, 18	19	18, 20	26	25, 28	19	18, 19
	Females	14	12, 15	13	11, 15	14	13, 15	15	14, 15	18	17, 19	24	22, 25	17	16, 17
Sweeteners	Males	3	2, 3	7	5, 8	8	7, 9	8	7, 9	5	5, 6	4	3, 4	6	6, 6
	Females	3	3, 4	4	4, 5	5	4, 5	4	3, 4	3	2, 3	2	2, 3	3	3, 4

* Without stews.

### Non-alcoholic beverages

For both sexes, the largest proportion of non-alcoholic beverages was water which accounts for approximately half of the consumption (Table 9). A quarter of the non-alcoholic beverages are coffee and green/black tea. The consumption of water is similar between both sexes. Women drank twice as much herbal/fruit tea per d than men. Consumption of soft drinks showed the opposite: the consumption in men was more than 2·5 times higher per d than women. The highest consumption of herbal/fruit tea was found in older age groups, whereas the consumption of fruit juice/nectars and soft drinks was lower in older participants. Both sexes met the dietary guidelines of the DGE (at least 1·5 l/d non-alcoholic beverage) very well.

**Table 9:** Mean daily consumption of non-alcoholic beverages (g) by sex and age groups (Mean values and 95 % confidence intervals)

Food group	Sex	14–18 years	19–24 years	25–34 years	35–50 years	51–64 years	65–80 years	Average
Mean	95 % CI	Mean	95 % CI	Mean	95 % CI	Mean	95 % CI	Mean	95 % CI	Mean	95 % CI	Mean	95 % CI
Water	Males	1086	1013, 1158	1294	1193, 1394	1169	1100, 1239	1187	1147, 1226	1111	1071, 1151	991	954, 1028	1137	1116, 1158
	Females	975	914, 1037	1106	1033, 1178	1153	1102, 1205	1201	1169, 1232	1193	1155, 1230	1057	1023, 1092	1140	1123, 1158
Coffee and tea (black/green)	Males	116	99, 132	295	257, 333	565	524, 606	708	682, 733	648	623, 674	534	515, 553	566	553, 578
Females	118	100, 136	272	240, 305	437	410, 464	610	591, 629	594	575, 613	488	472, 505	498	488, 507
Herbal/fruit tea	Males	82	66, 98	78	58, 99	105	87, 124	139	125, 154	180	163, 198	232	211, 253	149	142, 157
	Females	161	137, 184	228	196, 260	330	296, 364	333	313, 353	346	324, 368	346	324, 368	318	308, 329
Fruit juices/nectars	Males	488	441, 534	382	331, 432	379	334, 424	288	269, 308	221	202, 240	145	130, 159	285	274, 296
Females	403	368, 438	357	311, 403	321	291, 352	234	218, 249	181	166, 196	179	164, 193	245	236, 254
Soft drinks	Males	505	454, 556	471	441, 531	353	301, 405	206	187, 226	111	95, 127	41	33, 50	224	212, 236
	Females	260	225, 295	191	157, 225	118	101, 136	89	78, 100	37	29, 45	24	18, 30	88	82, 94
Other non-alcoholic beverages	Males	4	1, 6	11	3, 18	13	7, 18	17	12, 22	22	17, 27	24	19, 29	17	15, 19
Females	5	1, 10	3	0, 5	10	6, 13	17	14, 21	21	17, 26	24	19, 29	17	15, 19

### Alcoholic beverages

Men generally drank more alcoholic beverages than women (Table 10). Especially with regard to beer, men drank six times more than women. Additionally, concerning alcohol consumption differences were found between the age groups. Men aged 51–80 years and women aged 35–64 years drank more wine and sparkling wine compared to the other age groups.

**Table 10:** Mean daily consumption of alcoholic beverages (g) by sex and age groups (Mean values and 95 % confidence intervals)

Food group	Sex	14–18 years	19–24 years	25–34 years	35–50 years	51–64 years	65–80 years	Average
Mean	95 % CI	Mean	95 % CI	Mean	95 % CI	Mean	95 % CI	Mean	95 % CI	Mean	95 % CI	Mean	95 % CI
Beer	Males	162	140, 185	313	276, 349	227	202, 252	266	248, 284	279	258, 300	228	208, 247	253	244, 262
	Females	35	28, 43	50	40, 60	34	29, 39	37	34, 41	44	39, 50	36	31, 42	39	37, 41
Wine and sparkling wine	Males	5	4, 7	14	11, 17	35	30, 41	45	42, 49	65	59, 72	75	68, 83	48	45, 50
	Females	8	6, 9	28	22, 33	29	26, 32	47	43, 51	56	51, 60	33	29, 36	39	37, 41
Spirits and other alcoholic beverages	Males	19	13, 24	25	20, 31	8	6, 10	4	3, 4	4	3, 5	4	2, 6	7	7, 8
Females	14	10, 18	12	9, 14	3	2, 4	2	1, 2	2	1, 2	1	1, 2	3	3, 4

Among the different age groups, spirits and other alcoholic beverages like alcopops were consumed to the highest amount by young people (14–24 years).

### Socio-economic status

Concerning the SES there were no differences regarding the consumption of bread (Table 11). Participants with higher SES showed higher consumption of cereals and cereal products, but lower consumption of potatoes and potato products than those of lower SES. Men and women of higher SES ate more vegetables, fruit and fish, and consumed less meat and meat products, confectionery as well as fats and oils than participants with lower SES. Regarding the consumption of non-alcoholic and alcoholic beverages, there were different drinking habits between subjects with different SESs. Participants with higher SES drank more water as well as coffee and tea, but less soft drinks than those with lower SES. Furthermore, men with high SES consumed less beer but men and woman with high SES consumed twice the amount of wine than subjects with low SES.

**Table 11:** Mean daily food consumption (g) by sex and socio-economic status (SES) (Mean values and 95 % confidence intervals)

Food group		Sex		SES
Low (*n* 3235)	Medium (*n* 4767)	High (*n* 7369)
Mean	95 % CI	Mean	95 % CI	Mean	95 % CI
Bread	Males	180	175, 186	186	182, 191	186	182, 189
	Females	134	130, 137	135	132, 137	138	135, 140
Cereals and cereal products	Males	73	69, 77	77	74, 80	86	84, 88
	Females	61	59, 64	66	64, 68	77	76, 79
Potatoes and potato products	Males	98	94, 101	99	96, 101	87	85, 88
	Females	80	78, 82	74	73, 76	66	65, 67
Vegetables, mushrooms and pulses and products thereof	Males	219	210, 227	226	220, 232	241	237, 246
Females	224	218, 231	233	227, 238	260	255, 265
Fruit and fruit products (without juice)	Males	215	205, 226	229	220, 238	240	233, 247
	Females	269	259, 279	275	267, 284	290	282, 297
Milk, dairy products and cheese	Males	249	233, 265	273	260, 285	254	246, 262
	Females	239	228, 249	234	226, 241	238	232, 244
Meat, meat products and sausages	Males	152	147, 157	145	141, 150	133	131, 136
	Females	80	77, 82	76	74, 78	74	72, 75
Fish, fish products and seafood	Males	25	23, 27	27	26, 28	30	29, 31
	Females	21	20, 22	21	20, 22	23	23, 24
Eggs	Males	19	18, 20	17	16, 18	16	15, 17
	Females	13	12, 14	12	12, 13	12	12, 13
Fats and oils	Males	40	38, 41	39	38, 40	37	36, 38
	Females	29	28, 30	27	26, 28	26	26, 27
Soups	Males	64	59, 69	54	51, 57	48	46, 51
	Females	54	51, 58	45	42, 47	45	43, 47
Sauces and spicy ingredients	Males	47	44, 49	46	44, 48	44	43, 45
	Females	35	34, 37	34	33, 35	33	32, 34
Confectionery, total	Males	76	72, 80	73	71, 76	67	65, 69
	Females	69	66, 73	65	62, 68	59	57, 60
Water	Males	1054	1005, 1103	1133	1095, 1171	1185	1156, 1214
	Females	1081	1045, 1118	1095	1064, 1125	1224	1198, 1250
Coffee and tea (black/green)	Males	543	514, 573	521	499, 543	613	595, 630
	Females	477	458, 497	485	468, 502	524	511, 538
Soft drinks	Males	320	287, 354	250	227, 273	151	138, 163
	Females	123	107, 139	84	74, 95	66	59, 73
Beer	Males	284	258, 310	251	234, 267	238	227, 249
	Females	39	34, 44	37	33, 41	40	37, 43
Wine and sparkling wine	Males	32	27, 38	38	35, 42	63	60, 67
	Females	25	22, 29	32	29, 35	56	52, 59

## Discussion

The NVS II as a representative sample of the German population shows that the consumption of foods of plant origin is considerably below the dietary guideline values of the DGE^(^ ^2^ ^)^, while the consumption of foods of animal origin exceed these. For example, the consumption of vegetables was slightly more than half of the recommended amount; only one-seventh of the participants met these guidelines. The total consumption of meat, meat products and sausages among men was two-thirds higher than the upper range of the guidelines. The dietary guidelines of at least 1·5 litre of non-alcoholic beverages a day were met, the largest proportion of which was water (approximately half of the consumption of total non-alcoholic beverages).

As different dietary assessment methods were applied in other European national food consumption surveys conducted about the same time period, only a rough comparison of the food consumption is possible. For most of the food groups, a striking common pattern regarding the consumed amount could be observed. However, in Germany the consumption of vegetables was more than one-third higher than that in: the Belgian Food Consumption Survey 2004 of 3249 subjects aged 15 years or over (assessed by two 24-h recalls)^(^ ^16^ ^)^; the French Individual and National Food Consumption Surveys (2006–7) of 1922 subjects aged 18–79 years (assessed by a 7-d dietary record)^(^ ^17^ ^)^; the Finnish FINDIET Survey 2007 of 2039 subjects aged 25–74 years (assessed by one 48-h recall)^(^ ^18^ ^)^; and the Dutch National Food Consumption Survey (2007–10) of 2106 subjects aged 19–69 years (assessed by two 24-h recalls)^(^ ^19^ ^)^. The data of the Italian National Food Consumption Survey (2005–6) of 2831 subjects aged 18–97·7 years (assessed by a 3-d dietary record)^(^ ^20^ ^)^ and of the UK National Diet and Nutrition Survey (2008–9) of 431 subjects aged 19–64 years (assessed by a 4-d dietary record)^(^ ^21^ ^)^ showed a comparable consumption of vegetables for the adult population. Furthermore, in the UK and in Finland the mean consumption of meat and meat products was more than 1·5 times higher than in Germany, whereas in the other above-mentioned European countries similar amounts of meat were consumed. Additionally, for some food groups the European comparison revealed the highest consumption for Germany. The consumption of bread was nearly twice that of the UK and one-fifth more than that in the Netherlands. Compared to other European countries^(^ ^16^ ^–^ ^21^ ^)^, the consumption of fruit juices/nectars and beer was higher in Germany^(^ ^16^ ^–^ ^21^ ^)^.

For the NVS II participants, differences were found in food consumption between the sexes. Men showed a higher consumption of most of the food groups than women. On average they consumed two times more meat and soft drinks as well as six times more beer. In women, consumption of vegetables, fruit, and herbal/fruit tea was higher. Women in general showed a more favourable food choice in regard to the dietary guidelines of the DGE^(^ ^2^ ^)^. Other European food consumption surveys showed similar sex-specific food consumption, especially regarding consumption of meat and vegetables^(^ ^16^ ^–^ ^21^ ^)^. The higher absolute food consumption of men corresponds to their higher energy requirements compared to women. For most of the food groups food consumption calculated per 1000 kcal (4184 kJ) of total energy intake revealed no or only small sex differences (data not presented). After this adjustment for energy intake the consumption of vegetables and fruit was even higher and the consumption of meat was still less in women than in men.

Food consumption also differs between age groups. The amount of consumed foods and beverages per day was lower in elderly people: older men and women consumed less meat and meat products, fruit juice/nectars, soft drinks and spirits, whereas their consumption of fish, vegetables and fruit as well as herbal/fruit tea was higher than that of younger people. This means that women and elderly people make healthier food choices than men and younger adults. Similar trends were observed in other European national food consumption surveys^(^ ^16^ ^–^ ^18^ ^,^ ^20^ ^)^, in which the consumption of fish, vegetables and fruit was generally higher in elderly participants. Furthermore, the consumption of soft drinks, meat and meat products was higher in men and younger people.

In the present study, differences are found in food consumption regarding SES. The NVS II participants with higher SES consumed more vegetables and fruit, fish, water, coffee/tea and wine. Participants with lower SES ate more meat and meat products and drank more soft drinks and beer. Therefore, people with higher SES exhibit healthier food choices. Comparable dietary patterns regarding SES were found in other European studies. Results from a meta-analysis of European national food consumption surveys (including eleven studies from seven countries during the period 1985–99, participants in the individual surveys aged 18–85 years)^(^ ^22^ ^)^ revealed substantial differences between SES groups with respect to the consumption of vegetables and fruit. Subjects with higher SES consumed more vegetables and fruit. Furthermore, the data of three Dutch National Food Consumption Surveys (1987–8; 1992 and 1997–8) among a total of 6008 men and 6957 women aged 19 years and older showed that dietary intake among subjects with higher SES tended to be closer to the guidelines of the Netherlands Food and Nutrition Council. This finding was found quite stable throughout the decade of the study^(^ ^23^ ^)^.

The strength of the present study is the large population sample of 15 371 participants which is representative of the German population. Limitations of the present study should also be taken into account. Every dietary assessment method has different strengths and weaknesses regarding measurement of food consumption. A frequently mentioned limitation of the diet history method is the difficult cognitive task for study participants asked to recall food consumption of the previous 4 weeks^(^ ^24^ ^–^ ^26^ ^)^. This becomes more relevant as subjective influences like social desirability can affect responses. Surveys on intake of inhomogeneous food groups such as vegetables are especially affected by such subjective influences^(^ ^27^ ^)^.

A more general limitation of dietary assessment methods is underreporting. In the present study, 22 % of the NVS II participants were considered to be underreporters for whom the ratio of energy intake and resting energy expenditure was lower than the cut-off based on equations derived by Goldberg *et al.* ^(^ ^14^ ^)^ and adopted by Black^(^ ^15^ ^)^. Several national food consumption studies from other European countries show a similar level of underreporting^(^ ^16^ ^,^ ^17^ ^,^ ^20^ ^)^. A limitation of the comparison of the results of the present study with those of other European food consumption surveys is that different dietary assessment methods were used in the surveys which are very likely to affect the findings. This should be kept in mind when comparing food consumption surveys across Europe, and it underscores the need for synchronisation and standardisation of dietary survey methodologies. Implementation of the already existing guidelines on harmonised methods and protocols for national food consumption surveys^(^ ^28^ ^,^ ^29^ ^)^ would be the first step in this direction.

In conclusion, the results of the NVS II show that the German population in general consumes lower amounts of foods of plant origin and higher amounts of foods of animal origin than recommended. Substantial differences in food consumption exist between the different population groups (sex, age and socio-economic). Generally, women, elderly and people with higher SES tend to make healthier food choices and their food consumption is closer to the dietary guidelines. The consumption of most food groups in Germany is comparable to that in other European countries, whereas the consumption of bread, fruit juices/nectars and beer is highest in Germany.
